# Poly(etheretherketone)/Poly(ethersulfone) Blends with Phenolphthalein: Miscibility, Thermomechanical Properties, Crystallization and Morphology

**DOI:** 10.3390/polym13091466

**Published:** 2021-05-01

**Authors:** Adrian Korycki, Christian Garnier, Amandine Abadie, Valerie Nassiet, Charles Tarek Sultan, France Chabert

**Affiliations:** 1 LGP-ENIT-INPT, Université de Toulouse, 47 Avenue d’Azereix, 65016 Tarbes, France; adrian.korycki@enit.fr (A.K.); christian.garnier@enit.fr (C.G.); amandine.abadie@enit.fr (A.A.); valerie.nassiet@enit.fr (V.N.); 2 Prismadd Montauban, 2 Bis Rue Georges Courteline, 82000 Montauban, France; tarek.sultan@weare-aerospace.com

**Keywords:** polymer blend, miscibility, thermal transition, rheology, crystallization, morphology

## Abstract

Polyetheretherketone (PEEK)/polyethersulfone (PES) blends are initially not miscible, except when the blends are prepared by solvent mixing. We propose a route to elaborate PEEK/PES blends with partial miscibility by melt mixing at 375 °C with phenolphthalein. The miscibility of blends has been examined using differential scanning calorimetry (DSC) and dynamic mechanical analysis (DMTA). When adding phenolphthalein to PEEK/PES blends, the glass transitions are shifted inward as an indication of miscibility. We suggest that phenolphthalein acts as a compatibilizer by creating cardo side groups on PEEK and PES chains by nucleophilic substitution in the melted state, although this condensation reaction was reported only in the solvent until now. In addition, phenolphthalein acts as a plasticizer for PES by decreasing its glass transition. As a consequence, the PEEK phase is softened which favors the crystallization as the increase of crystalline rate. Due to aromatic moieties in phenolphthalein, the storage modulus of blends in the glassy region is kept identical to pure PEEK. The morphological analysis by SEM pictures displays nano- to microsized PES spherical domains in the PEEK matrix with improved PEEK/PES interfacial adhesion.

## 1. Introduction

Polymer blends play a central part in the development of new materials with tailored properties. Indeed, the interest arises from the ability to tune their morphology and physical properties. Polyaryletherketones (PAEK) offer the best performance in terms of mechanical properties, chemical resistance, and durability among all thermoplastics. However, cutting-edge applications require multifunctional materials. Ideally, one material should fit all the targeted properties. Blending two polymers may be the easiest option to design such materials with controlled morphology. In most cases, thermoplastics are not miscible, and the resulting blends display nice structures such as droplets dispersed in a main continuous phase, from spherical to elongated domains up to co-continuous phases when the phase ratio is close to 1. Although such structures constitute beautiful and refined images to entertain scientists, these polymer blends are typically described as unstable phase morphologies. Even worse, without interfacial interaction between both phases, these systems demonstrate poor mechanical properties. The lack of adhesion at the contact area is the place where microcracks appear, and from there, they propagate inside the material up to its complete fracture. Compatibilization makes the system more stable and better-blended phase morphology by creating interactions between the two previously immiscible polymers. The compatibilization not only enhances the mechanical properties of the blend, but also yields properties that are generally not attainable in a single pure component.

Polyaryletherketones are only miscible with polyetherimide (PEI) as attested by numerous works [1,2,3,4,5]. Nevertheless, some other amorphous polymers look like promising options to enhance the properties of PAEK-based blends.

Taking into consideration polyetheretherketone (PEEK) and polyethersulfone (PES) blends, some works report that they are partially miscible when prepared by solution mixing. For instance, it was found that they were compatible when prepared by solution blending in diphenyl sulfone at 310 °C, with a single-glass transition temperature reported by Yu et al. [6] for various compositions. The authors noted that the films processed by compression moulding at 310 °C have been found with one-glass transition temperatures for each composition. However, when the films were processed at 350 °C, two glass transition temperatures were measured for each blend. It indicates that phase separation occurs in this case. Therefore, it means that the phase diagram would reveal a low critical soluble temperature behavior with the cloud point located between 310 and 350 °C when the fraction of PES is in the range from 70 to 30 wt %. In addition, Ni [7] prepared the blends by solvent mixing in sulphuric acid. The 50/50 PEEK/PES blend demonstrates two glass transition temperatures at 146 and 230 °C, close to those of pure PEEK and PES, respectively. The presence of two separated phases was concluded from the stability of the glass transitions. Moreover, the crystallization peak at 180 °C and the melting point at 333 °C are the same compared to those of the neat polymers.

Besides, PEEK and PES blends have been prepared by melt mixing at 355 °C, and they have been characterized by Malik [8]. They take a single-glass transition temperature for each blend as a criterion for miscibility, so they concluded that these blends may be miscible. They suggested that the miscibility in PEEK/PES blends stems from the interaction of the carbonyl group of PEEK and the highly polarizable aromatic sulfonate structure of PES. The degree of crystallinity and the melting peak significantly decreased with the amount of PES in the blend, from 42% to 27% and from 342 to 325 °C, respectively. Therefore, it looks that the crystallization of PEEK is hindered by the presence of PES chains. Similar results have been reported by Arzak et al. [9] on a 50/50 weight ratio PEEK/PES blend after a quenching treatment.

To sum up, it appears that mixing PEEK/PES forms immiscible blends, even if the presence of the solvent used to solubilize both phases could favor their compatibility to create partially miscibility of blends. PEEK/PES blends have been mainly prepared by solution blending; however, a few works report that they can be also obtained by melt mixing.

Taking a look at the mechanical properties, according to Yu et al. [6], the elastic modulus of PEEK dropped dramatically from 3000 MPa at room temperature to 1200 MPa in the range of 150–180 °C, while for PES the modulus decreased from its glass transition at 240 °C. The addition of PES can remarkably improve the elastic modulus of PEEK up to 240 °C. For the 40/60 PEEK/PES blend, the elastic modulus was about 10 times that of pure PEEK at 180–220 °C. Malik [8] has reported that tensile strength and tensile modulus also significantly increased with the addition of PES from 77 to 135 MPa and from 2550 to 4650 MPa, respectively. An irregular enhancement of mechanical properties was observed with increasing the PEEK amount to the maximum amount of around 60%. Then, the results of tensile strength and Young’s modulus followed the linear values predicted by the rule of blending. This improvement is explained by the morphology of the blends: the 20/80 PEEK/PES blend showed spherical and well-distributed PEEK domains inside the PES matrix. This morphology arises from phase separation. However, good interfacial adhesion between both components is claimed by Malik [8]. PEEK and PES are not miscible from a thermodynamic point of view, but some compatibility may be achieved for particular blending conditions. Compatibility stems from specific interaction between sulfonate groups of PES and PEEK backbone. Hence, the mechanical properties are higher than those expected from the additivity rule.

Thus, even if PEEK/PES blends are thermodynamically immiscible, the affinity of both phases could be improved to reach the compatibility and stability of these systems. Therefore, modifying one of the polymer backbones with side groups or adding another component is necessary. Ternary blends may be relevant as hinted in the previous sections. Indeed, the third component may act as a link between two immiscible materials, provided it is miscible with both. One of the mechanisms for the compatibilization of immiscible blends is the addition of segmented block copolymers of the type (A-B)_n_ consisting of segments chemically comparable to the parent homopolymers [10]. Properties of segmented block copolymers in PEEK/PES blends were studied by Hoffmann et al. [11]. Summarizing the mechanical properties, improved strength and toughness were reached, but the effectiveness of the segmented block copolymers was hindered by the absence of phase separation between the PEEK and polyethersulfone segments. It is expected that phase-separated segmented PEEK/PES block copolymers result in a higher compatibilization effect in the blends. Mitschang et al. [12] reported a blend of PEEK/PEI/PES used as composite matrices. The tensile strength, modulus, and elongation have been reported as 92 MPa, 3400 MPa, and 5.4%, respectively, while the tensile strength, modulus, and elongation for neat PEEK were 100 MPa, 3500 MPa, and 5.0%, respectively, the tensile strength, modulus, and elongation for neat PEI were 105 MPa, 3200 MPa, and 6.0%, respectively, and the tensile strength, modulus, and elongation for neat PES were 90 MPa, 2700 MPa, and 6.7%, respectively. It can be concluded that three different polymers may be successfully blended while keeping acceptable mechanical properties.

An option proposed first by Zhang and Zeng [13] to improve the PEEK/PES miscibility is the incorporation of cardo side groups. Usually, cardo side groups are added into a polymer matrix during the aromatic nucleophilic substitution reaction. It can be obtained from 4.4′-difluorophenyl sulfone or difluorobenzophenone in solvent mixing (consisting of dimethylformamide, toluene, and potassium carbonate) at 145 °C for 8 h. This procedure is suitable to link cardo side groups with PES and PEK [14].

**Figure 1:**
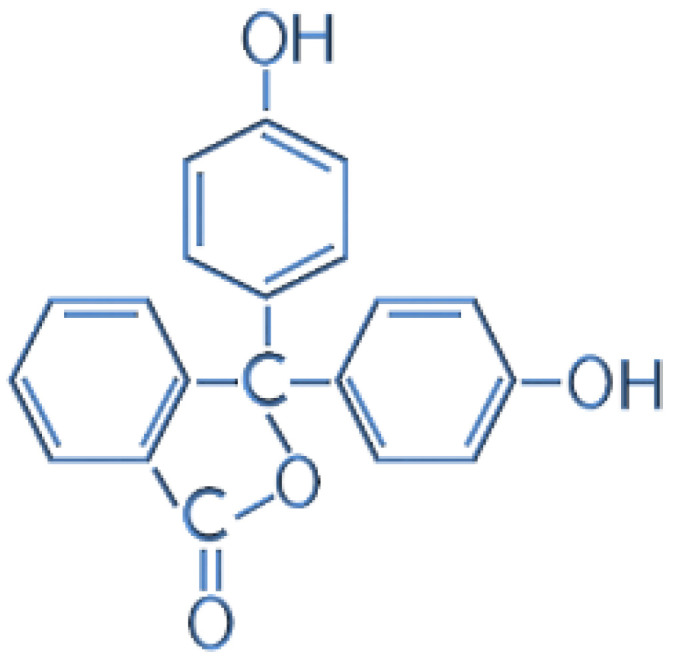
Chemical structure of phenolphthalein.

Blends of PEEK/PEK-C (PEK with cardo side groups) or PEEK/PES-C (PES with cardo side groups) have high operating temperatures and good mechanical properties. They are partially miscible with a slower crystallization, a lower melting point, and a lower PEEK degree of crystallinity [13,15]. In addition, cardo side groups may be created by phenolphthalein. Polymers with cardo side groups are a subgroup of polymers where carbons in the backbone of the polymer chain are also incorporated into ring structures, as shown in Figure 1. However, this method requires solvents, so it is not environmentally friendly. That is why we propose a way to elaborate PEEK/PES blends with phenolphthalein by melt mixing, which is a solvent-free process.

To our knowledge, it is the first time that PEEK/PES/phenolphthalein blends are reported. Moreover, in the previous works on PEEK/PES blends, they were prepared by solvent mixing, while we prepare our samples by blending at the melted state. In this work, two grades of PES with different molecular weights have been chosen. Our main goal is to obtain miscible PEEK/PES blends while keeping mechanical strength in the glassy state comparable to those of PEEK.

The miscibility, thermomechanical properties, and crystallization of PEEK/PES blends have been examined by differential scanning calorimetry (DSC) and dynamic mechanical analysis (DMTA). Their morphologies have been evaluated by scanning electron microscopy (SEM) to make a correlation between the morphology and the properties of the blends.

## 2. Materials and Methods

### 2.1. Materials

The polymers were commercial products of which properties are shown in Table 1. The selected PEEK was PEEK 450G purchased from Victrex. PESs were Ultrason E1010 and Ultrason E3010 from BASF. The effect of chain lengths on the miscibility was studied with PES 1010G representing short chains, while PES 3010G had longer chains. The molecular weight of PES has been provided by the supplier datasheet. It can assume that a blend with the polymer of longer chains would give an immiscible blend or partially miscible, but with high mechanical rigidity, while a blend with shorter chains would result in a material with better miscibility but lower rigidity.

Phenolphthalein (C_20_H_14_O_4_) from Fisher Scientific in powder form is slightly soluble in water and usually soluble in alcohol. It is a weak acid with a melting point of 260 °C, which can lose a proton (H^+^) in a solution.

### 2.2. Methods

#### 2.2.1. Blends Preparation

PEEK and PES were blended in mass ratios of 100/0, 90/10, 80/20, 70/30, and 0/100 wt % separately. Before blending, each polymer was dried in a vacuum oven at 120 °C for at least 48 h. Melt mixing was carried out in a parallel twin-screw micro-extruder, Process 11 from ThermoFisher. Before extrusion, the two chosen polymers were physically blended in a beaker in appropriate weight ratios in small batches of 10 g and then fed into the feeder of the twin-screw extruder. The temperature of the rotors and die zone were kept at 350 °C, and the rotor speed of 70 rpm was used for all blend compositions. In our conditions, the residence time (time spent inside the extruder until it came out of the die) inside the blending zone was measured to be 2 min and 20 s. Due to the unavailability of the twin-screw micro-extruder Process 11, the blends with phenolphthalein were prepared with another twin-screw micro-extruder, Xplore MC 15 HT. The temperature was set at 375 °C, and the screws speed was fixed at 100 rpm. The residence time was 2 min and 30 s. The parameters (i.e., shear rate, temperature, and residence time) are kept as close as possible to those set in Process 11. This residence time was short enough to prevent degradation. Each blend was extruded into filaments through a 2 mm-diameter die. The filaments were cooled under air. Further, the filaments were chopped into small granules.

The PEEK/PES blends with a compatibilizer were separately blended in ratios of 100/0/10, 90/10/10, 80/20/10, 70/30/10, and 0/100/10 (wt %), meaning that the phenolphthalein was added as a 10% volume over the entire blend. This 10% ratio was chosen according to the relevant literature on the compatibilization of polymer blends. Phenolphthalein significantly decreased the torque during mixing, and the melt looked less viscous compared to those of other blends. Preliminary measurements revealed that the complex viscosity of blends with phenolphthalein was 10 times lower than those without phenolphthalein. The compositions of all samples prepared are listed in Table 2.

#### 2.2.2. Preparation of Samples

For further testing, the blends were processed by compression moulding. A hydraulic press LAB 800P PEI from Pinette Emidecau Industries was used for preparing 2 mm-thick plates. The granules were dried in a vacuum oven at 150 °C for at least 3 h. A steel mould with a 149 mm × 76 mm × 2 mm cavity was used. Granules of blends were kept in the cavity between two steel foils and placed between the plates of the hot press, which was preheated at 200 °C. Next, the temperature was increased at a speed of 10 °C·min^−1^ up to 360 °C. A pressure of 50 kN was applied at 360 °C for 5 min. Then, still under the pressure, the moulded samples were cooled down until 200 °C at the speed of 4 °C·min^−1^ under pressure. Finally, the plates were separated from steel foils and left at room temperature to slowly cool down.

#### 2.2.3. Experimental Methods

The apparatus used for thermal analysis was DSC Q200 (Thermal Analysis Instruments). The measurements were carried out under a nitrogen flow of 50 mL·min^−1^ and at heating and cooling rates of 10 °C·min^−1^ from ambient temperature to 380 °C. A mass of approximately 10 mg was placed in sealed aluminium pans. The specimens were previously dried for at least 3 h at 150 °C. The glass transition temperature (*T*_g_), crystallization temperature (*T*_c_), and melting temperature (*T*_m_) were determined with an accuracy of approximately 1%. The apparent degree of crystallinity (*X*_c_), with the accuracy of more or less 5%, was calculated for the weight fraction of the crystalline phase by the following equation:

*X*_c_ = [(Δ*H*_m_/wt.%PEEK)/Δ*H*_th_] × 100%, (1)

where Δ*H*_m_ is the melting enthalpy (J·g^−1^) and Δ*H_t_*_h_ is the theoretical melting enthalpy of the 100% crystalline phase (J·g^−1^) equaling to that of PEEK (130 J·g^−1^) [18]. In the blends, only the PEEK phase can crystallize, and PES is an amorphous polymer. The degree of crystallinity of PEEK in blends was calculated by considering the PEEK ratio in each blend composition using Equation (1).

The thermomechanical properties were performed by dynamic mechanical analysis with an ARES LN2 rheometer from Rheometrics. The 45 mm × 10 mm × 2 mm rectangular specimens were dried for at least 3 h at 150 °C before testing. The tests were carried out in torsion mode at a frequency of 1 Hz within the viscoelastic linear domain at a heating rate of 3 °C·min^−1^ from 25 to 325 °C. The accuracy was considered at approximately 2%. The storage modulus (E’) characterizing the elastic behavior of the blend, the loss modulus (E”) characterizing the viscous behavior of the blend, and the loss factor (tanδ = E”/E’) were determined.

The morphology of blends was studied on cryogenic fractured surfaces using SEM performed with Inspect S by the FEI Company. The images were registered in a gradient vacuum with an accelerating voltage of 10 kV and in analysis mode with a magnification of ×10,000.

## 3. Results and Discussion

### 3.1. PEEK/PES Blends

#### 3.1.1. Miscibility by DSC and DMTA

**Figure 2:**
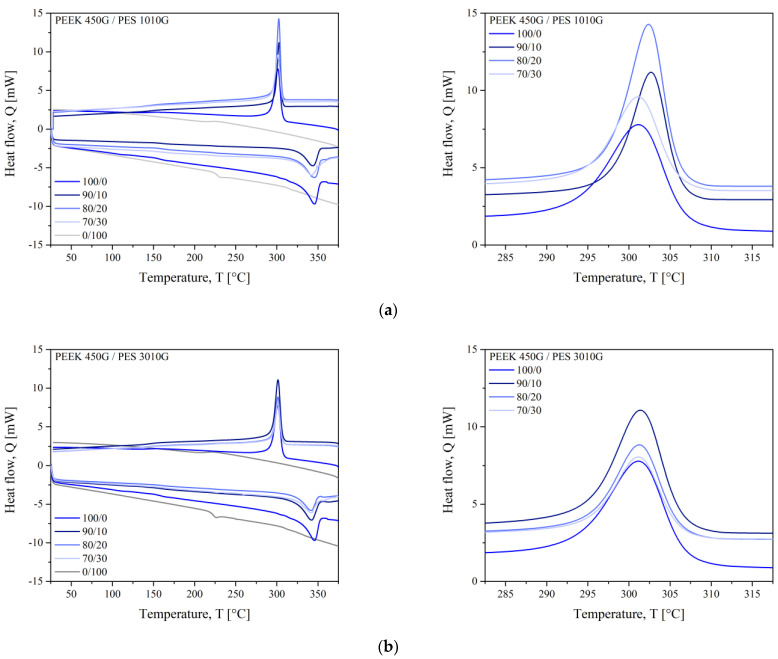
The differential scanning calorimetry (DSC) thermograms for PEEK/PES 1010G blends (**a**) and PEEK/PES 3010G blends (**b**).

Probing the glass transition temperature (*T*_g_) as a diagnostic aid for determining compatibility or incompatibility of polymer blends is now an accepted criterion [19]. Typical thermograms obtained for PEEK/PES blends are shown in Figure 2. The glass transitions are reported in Table 3, and the curves are provided in Supplementary Information. Firstly, each polymer was processed separately according to the same extrusion conditions. For PEEK, the glass transition was shifted from 147 to 159 °C due to the processing conditions. It is known that PEEK is sensitive to thermo-oxidative degradation and its macromolecular chains undergo recombination and cross-linking under heat. This could explain the stiffness brought to PEEK chains when extruded. On the contrary, for PES, the glass transition of each grade went down from 232 to 228 °C and from 236 to 223 °C for 1010G and 3010G, respectively. Again, the processing conditions were responsible for these slight changes.

In the blends, two glass transitions associated to PEEK at 159 °C and to PES 1010G at 228 °C on the one side and those related to PES 3010G at 233 °C on the other side are measured for the 90/10 PEEK/PES blends. Comparing to those of individual polymers, the *T*_g_ values of these blends were unchanged. It indicated the immiscibility of the blends for all compositions. The glass transition of PEEK in the blends was higher than those of virgin PEEK at 147 °C, because PEEK was processed by extrusion to be consistent with the thermomechanical history of blends.

The melting endotherm (*T*_m_) of PEEK was observed with a maximum temperature of around 345 °C. On cooling, an exothermic peak (*T*_c_) was seen at around 300 °C which corresponded to the crystallization of PEEK. When compared with those of pure PEEK (still extruded in the same condition) and PEEK/PES 1010G blends in Figure 2a on the right, the crystallization onset temperature was the same for all blend compositions at 300 °C and this crystallization onset temperature was very close to that of pure PEEK. The shape and the width of the melting and crystallization peaks were similar to those of neat PEEK, which indicated that the vicinity of PES chains did not change the dimensions of crystalline structures of PEEK. The change in the degree of crystallinity with the PES ratio is discussed in the next section.

**Figure 3:**
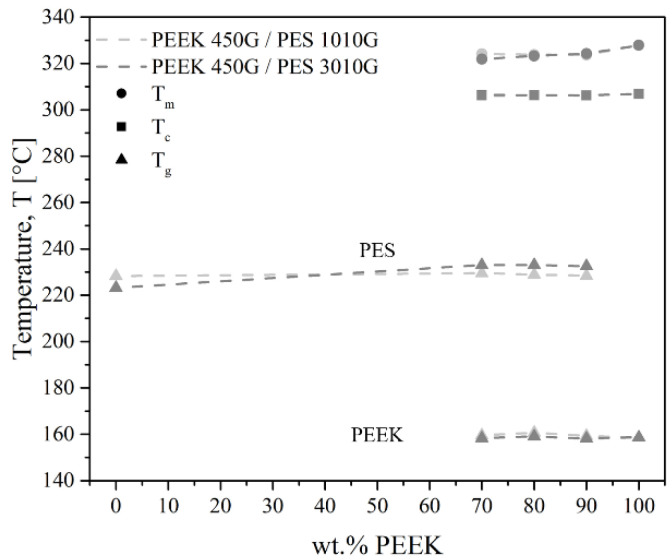
Thermal transitions of PEEK/PES blends by DSC.

All the thermal transitions measured for PEEK/PES blends are presented in Figure 3. Neither the crystallization temperature of PEEK nor the melting temperature was modified in the blends. The blends exhibited two *T*_g_, indicating the immiscibility of the two phases. Moreover, there was no effect of chain length. The thermal transitions were the same for both PES grades.

**Figure 4:**
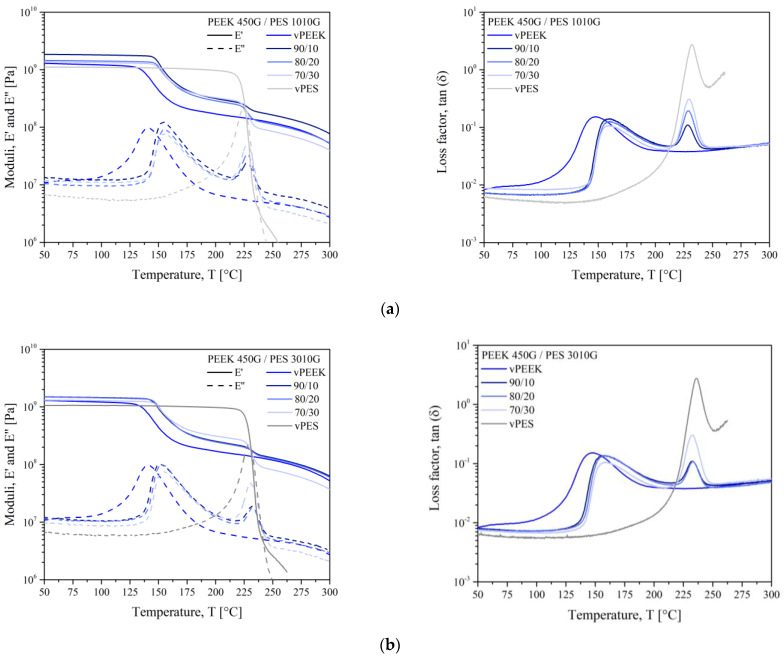
The dynamic mechanical analysis (DMTA) thermograms for PEEK/PES 1010G blends (**a**) and PEEK/PES 3010G bends (**b**).

The thermomechanical responses for PEEK, PES, and their blends are presented in Figure 4. The dynamic mechanical analysis gave information on the mechanical properties and the compatibility of the blends. The value of the storage modulus (E’) signified the stiffness of the material. The curves of E’ and the loss modulus (E”) for PEEK and PES were typical of the behavior of their semi-crystalline and amorphous structures, respectively. The curves for PEEK exhibited three distinct regions: a high-modulus glassy region where the segmental mobility was restricted, a transition zone where a substantial decrease in the storage modulus with the increase in temperature, and a rubbery region (the flow region) where a drastic decay in the modulus with temperature was seen. The storage modulus curve of PES showed the typical behavior of an amorphous polymer, which migrated from an energy-elastic to an entropy-elastic state after reaching the glass transition temperature. Both polymers displayed a glass transition (*T*_α_) that represented the onset of molecular motion in the amorphous region. In PES, the mechanical strength E’ decreased right after the glass transition. The rubbery plateau was the result of entanglements. The width of this rubbery plateau depended on the molecular weight between entanglements (*M*_e_). Since no rubbery plateau seemed to appear at *T* > *T*_g_ for PES, it indicated that the number of entanglements was low and the molecular weight of PES could be the same order of magnitude as its molecular weight between entanglements.

However, in PEEK, the decline in storage modulus was around 50% compared to those at room temperature, due to the contribution of the rigidity of the crystalline structure.

When blend components were immiscible, each component exhibited its own unperturbed relaxation process. It is known that the signature of miscibility in blends is when the glass transition temperatures tend to get closer to each other. In Figure 4, PEEK and PES kept their own glass transitions. As expected, this indicated the presence of two pure phases in the blends. It is worthy to comment on the curves of the virgin PEEK reported in Figure 4 as vPEEK. As explained above, the chemical structure of PEEK was modified by the extrusion step. For this reason, the curves for virgin PEEK were far from those of blends: the storage modulus was lower in the whole temperature range and the maximum of the loss modulus appeared at a lower temperature. Additionally, the E” peak was broader and more symmetrical compared to those of PEEK in blends. Indeed, the extrusion step could extent the average PEEK chain length and change its polydispersity. The shape of the E” peak, even if related to the amorphous phase, broadened and shifted towards higher temperatures, when the crystalline rate was higher, as attested by Illers and Breuer’s work in 1963 [20]. Another explanation is the migration of PES chains inside the amorphous phase of PEEK. Indeed, when looking at the shape of E’’ peaks and loss factor peaks, it is clear that the peaks lost their symmetrical shape when the polymers were blended. The E’’ peak shape for PES was conserved on the right side (longest chains), while it broadened on the left side (shortest chains). The inverse shape was observed for PEEK. It meant that the shortest chains of PES could migrate inside the amorphous PEEK phase, broadening the molecular distribution of the PEEK-rich phase.

The two glass transitions were obvious for all blend compositions, and they all showed clear dependences on PES content. The storage modulus, presented from 50 °C to 300 °C, increased for all the blends compare to neat thermoplastics: the storage modulii at 50 °C is 1300 MPa and 1100 MPa for PEEK and PES, respectively. The highest modulus of 1850 MPa has been obtained for the 90/10 wt % blend of PEEK and PES 1010G. Once heated above the glass transition of PEEK, the storage modulus dropped to a significantly lower level for each composition. The high glass transition temperature of PES above 225 °C retained the relevant stability of the blends in the range 180 to 220 °C: all the blends showed a storage modulus of around 300 MPa. As expected, the blends were immiscible for all the compositions.

Then, Figure 4 on the right presents the loss factor versus temperature curves for PEEK/PES blends. It can be seen the characteristic immiscible behavior with two peaks corresponded to the glass transition temperature of PEEK and PES phases. The trends noticed for loss modulus were observed for loss factor (tan δ). The *T*_α_ values obtained for blends did not change with PEEK/PES fraction. From our results, PEEK and PES were clearly immiscible. However, some authors [21] declare the existence of PEEK-rich and PES-rich phases in each blend composition, as observed by Nandan et al. [22] with differences of glass transition temperatures for PEEK from 164 to 171 °C and PES from 235 to 226 °C in the composition of 75 wt % of PEEK.

#### 3.1.2. Crystallization of Blends

**Figure 5:**
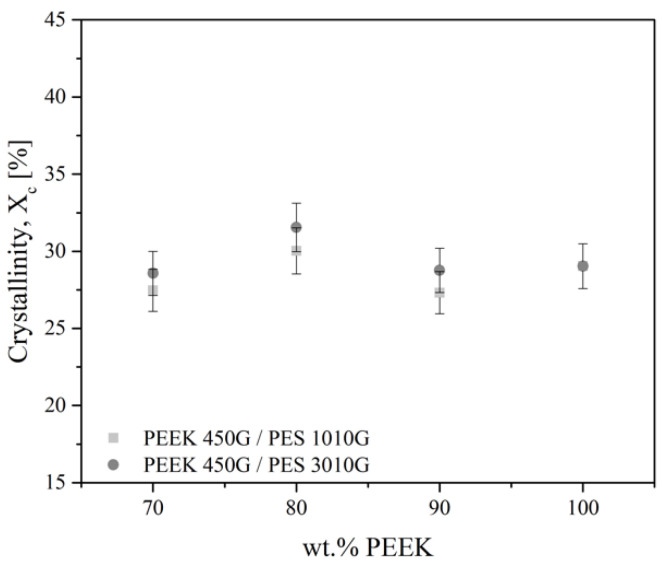
Degree of crystallinity (±5%) for PEEK/PES blends.

Then, the change of crystallinity of PEEK inside the blends was evaluated. The degree of crystallinity (*X*_c_) of PEEK in the blends is presented in Figure 5. The degree of crystallinity remained close to the degree of crystallinity of pure PEEK in the blends with PES. The degree of crystallinity is 29% for pure PEEK, 27% for the PEEK/PES 1010G blends (70/30 wt %), and 29% for the PEEK/PES 3010G blend (70/30 wt %). These values are similar considering the uncertainty. As expected, the degree of crystallinity did not significantly change in the immiscible blends. It looks that there was no significant effect of adding longer or shorter PES chains on the crystallization of PEEK.

Considering our results, Arzak et al. [9] concluded that the crystallization-melting behaviour of PEEK is little affected by the presence of PES in the blends. For instance, the degree for the 70/30 PEEK/PES blend is 16.6%, calculated from density measurements. However, this value seems to be relative to the whole sample, whereas when we reported it based on the PEEK phase only, the degree of crystallinity is 24%. Moreover, the link with our results obtained by DSC is not possible as attested by the work done by Doumeng [23] on the comparison of DSC, density, and other techniques to evaluate the crystalline rate of PEEK. Surprisingly, Malik [8] has reported that PEEK/PES blends containing less than 40 wt % of PEEK are fully amorphous, and beyond this concentration, PEEK can crystallize in the blends, as measured by DSC. The same authors reported that the degree of crystallinity highly decreases when the PES ratio is increased. As an explanation, they proposed that PEEK/PES blends are partially miscible due to some specific interactions between the two polymers, possibly the interaction of the carbonyl groups of PEEK and the sulfonate groups of PES. However, this phenomenon is not effective in our case.

### 3.2. PEEK/PES Blends with Phenolphthalein

#### 3.2.1. Miscibility by DSC and DMTA

**Figure 6:**
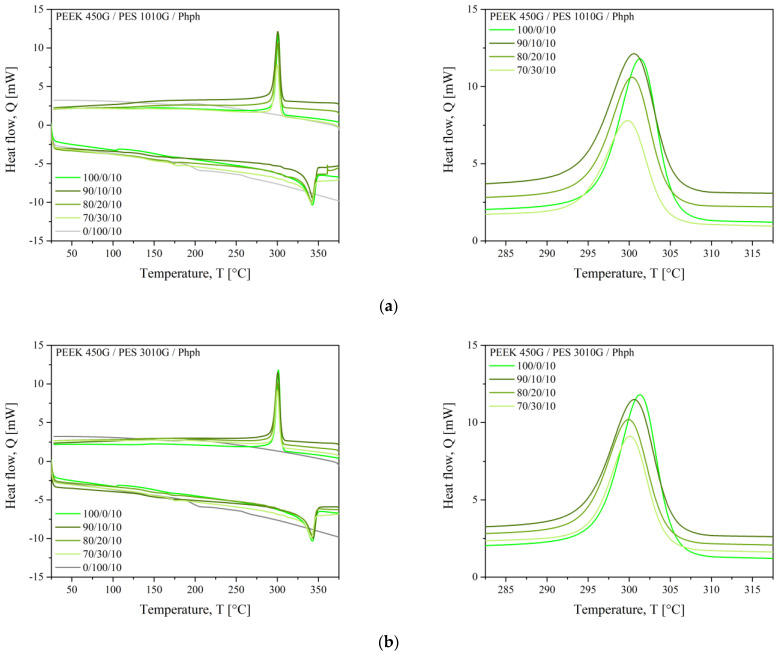
The DSC thermograms of PEEK/PES blends with phenolphthalein (**a**) 1010G (**b**) 3010G.

The DSC thermograms of blends with phenolphthalein are presented in Figure 6. First, every single polymer has been blended separately with phenolphthalein to probe their interaction. The glass transition temperatures of PEEK and PES with phenolphthalein are reported in Table 4. The *T*_g_ of PEEK slightly decreased when adding phenolphthalein, ranging from 159 to 151 °C. Identically, phenolphthalein brought more mobility to PES chains, with the *T*_g_ of PES 1010G from 228 to 191 °C and the *T*_g_ of PES 3010G from 223 to 195 °C.

The changes in glass transition temperature could be associated with cardo side groups grafted on the end groups or with a plasticizing effect by van der Waals and hydrogen bonds without chemical reaction. The two hypotheses are discussed below.

**Figure 7:**
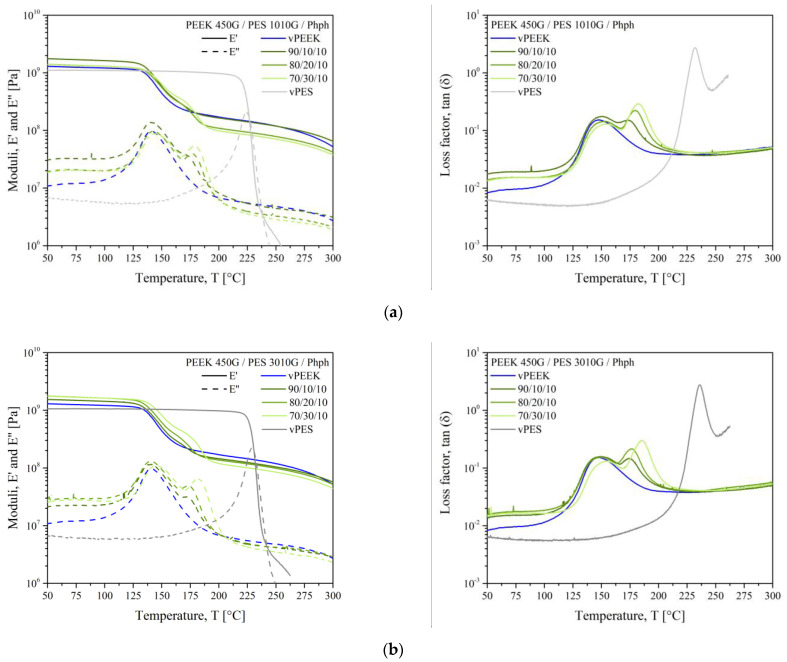
The DMTA curves of PEEK/PES blends with phenolphthalein: (a) 1010G; (b) 3010G.

Let us focus on the PEEK/PES blends with phenolphthalein. Their DSC thermograms are presented in Figure 6, and the thermomechanical responses for PEEK, PES, and their blends with phenolphthalein are presented in Figure 7. Still, two glass transitions were visible, but they are shifted inward, as reported in Table 4. Let us look at the effect on each polymer. Firstly, for PEEK, the *T*_g_ was increased from 147 °C (virgin PEEK) to 151 °C in the PEEK/phenolphthalein blend and to 154 °C for 70/30/10 blends. Comparing our results with the same thermal history, it seems the evolution of the structure of PEEK in a thermo-oxidative environment is prevented in the presence of phenolphthalein. Further experiments are necessary to confirm this perception.

However, the effect of phenolphthalein was greater on the glass transition of PES, for which the glass transition temperature lowered from 191 °C for pure PES to 173 °C for 90/10/10 blends with PES 1010G. Identically, it went from 195 °C for pure PES to 174 °C for 90/10/10 blends with the longest macromolecular chains of PES 3010G.

The E” peaks approach one with each other up to 25 °C for blends with 10 wt % of PES 3010G. This shift of glass transition agrees with our DSC results. The E’’ peaks were broadened compared to those of pure polymers, showing the widening of the molecular weight distribution when mixing with phenolphthalein. The storage modulus curves of blends with PES 3010G were much closer to the shape of the pure PEEK curve, because the composition of blends was mainly PEEK.

Let us evaluate the effect of phenolphthalein on PES. For that, we considered the hypothesis that the PEEK has no interaction with neither PES nor phenolphthalein. The relevant control parameter is then the PES/phenolphthalein ratio which changes for each blend composition. For instance, in the 90/10/10 blend, the ratio is about 50 wt %, and in the 0/100/10 blend, the ratio is 10 wt %.

**Figure 8:**
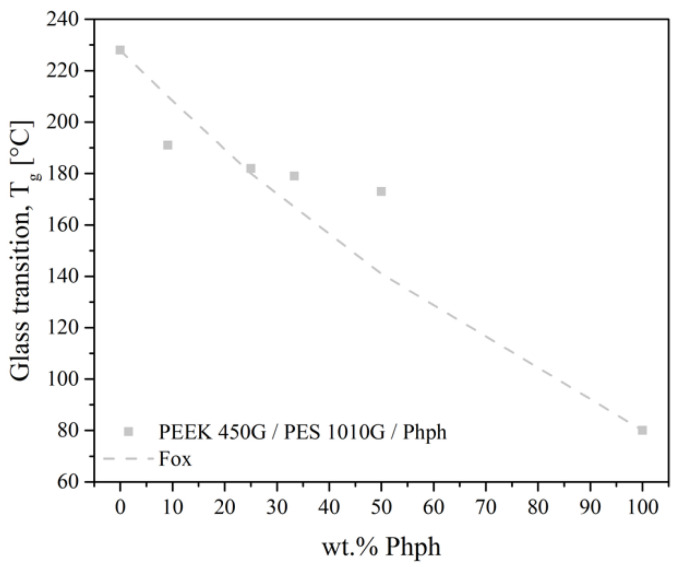
Glass transition of PES versus phenolphthalein composition (wt % Phph) in the phenolphthalein/PES blends. For instance, 90/10/10 corresponds to 50 wt %. 0 wt % indicates pure PES, and 100 wt % represents pure phenolphthalein.

The *T*_g_ of each blend progressively changes between the *T*_g_’s of the homopolymers in the amorphous blends, according to the Fox equation within an experimental error [24]:

1/*T*_g,mix_ = Σ_i_w_i_/*T*_g,i_, (2)

where *T*_g,mix_ and *T*_g,i_ are the glass transition temperatures of the mixture and the components (K), respectively, and *w*_i_ is the mass fraction (%) of component i. The glass transition of PES is plotted in Figure 8 to examine the effect of phenolphthalein. The curve corresponds to the Fox equation [24]. The latter fits well with the experimental data when there are no strong inter- or intramolecular interactions [25]. The Fox equation considers a synergetic effect of components on the local motion, which can be identified as an energetic barrier (activation energy) influenced by the local environment. The *T*_g_ of PES/phenolphthalein mixture (0/100/10 wt %) was below the Fox curve, indicating that a part of the phenolphthalein probably plasticized the PES thanks to weak interactions and another part was free in the mixture because the concentration of phenolphthalein was too high to interact with PES. On the contrary, when blended with PEEK, i.e., in the ternary blends, the *T*_g_ of PES was higher than the Fox curve. This result suggested that the interaction of phenolphthalein with PES is intensified in presence of PEEK.

As a reminder, in the previous section, the glass transition of PES was not modified in the PEEK/PES blends without phenolphthalein. The phenolphthalein interacted preferentially with PES, regardless of the chain length, giving more mobility to the PES macromolecules. As the shifting of *T*_g_ inward is often associated with miscibility, the phenolphthalein could be considered as a compatibilizer in these systems.

However, as any small molecule, phenolphthalein could be associated with the plasticization of PES, to create a free volume and to reduce the friction of macromolecules on themselves. The plasticizer makes the polymer softer and more flexible, and it increases its plasticity and decreases its viscosity, due to the associated free volume. The action of plasticizer often results from the creation of hydrogen bonds with hydrophilic parts of polymer. The plasticizing effect is effective for PES, but it is less significant for PEEK, for which *T*_g_ decreases by a few degrees only.

**Figure 9:**
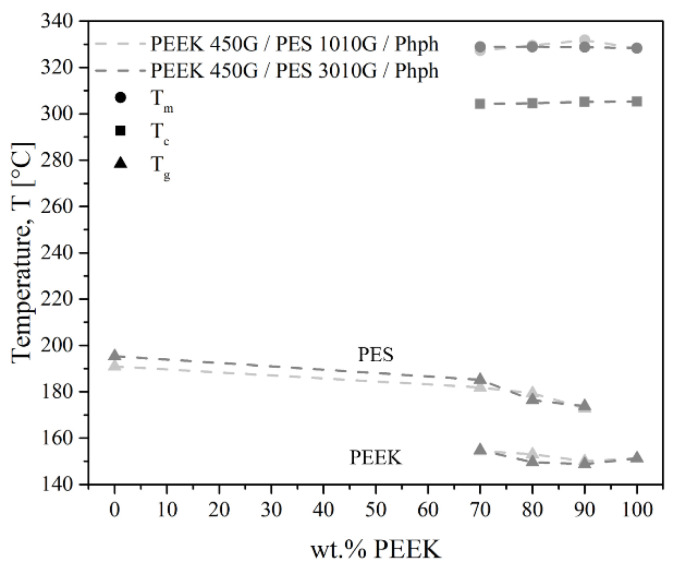
Thermal transitions of PEEK/PES blends with Phph by DSC.

All the thermal transitions measured for PEEK/PES blends with phenolphthalein are presented in Figure 9. The *T*_g_ shift was visible, whereas the crystallization and melting temperatures were unchanged. Neither the maximum values of the peaks nor their shapes were modified. We assumed that the phenolphthalein interacted only with the amorphous PEEK phase whereas the crystalline phase was not modified, so that the crystalline structure and dimensions of PEEK in blends stayed identical as it was in pure PEEK. Nevertheless, the effect on the crystalline rate of PEEK is discussed below.

**Figure 10:**
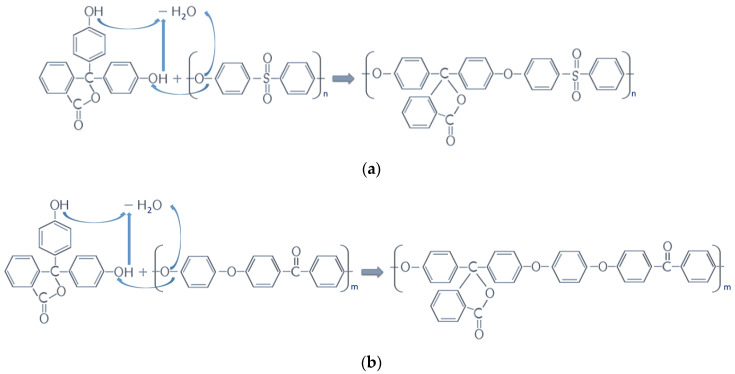
Chemical reaction of phenolphthalein with PES (**a**) and PEEK (**b**).

Moreover, due to the high reactivity of phenolphthalein, which contains labile hydrogen, chemical bonds could be created. Indeed, PEEK is synthesized by a nucleophilic substitution obtained by polycondensation between 200 and 400 °C. The monomers are biphenyl(hydroquinone) and a fluorinated aromatic compound in a polar aprotic solvent (diphenyl sulphone). Fluorinated derivatives are preferred for this synthesis because of their better reactivity and their higher electronegativity than those of chlorinated derivatives [26]. Thus, some PEEK chains could be ended by fluorinated groups. The latter is subjected to react with the labile proton of phenolphthalein by nucleophilic substitution. The chemical scheme is proposed in Figure 10. Thus, the so-grafted phenolphthalein, named cardo side groups, extends the macromolecular chains of PEEK and PES. In addition, as the reaction is possible with both PEEK and PES, phenolphthalein would act as a compatibilizer by connecting PEEK and PES in some chains.

According to the same chemical route, Zhang and Zeng [13] have prepared PEEK/PES-C blends, in which PES-C means PES with cardo side groups. PES-C is not commercially available, but its synthesis is reported in a patent [27]. The preparation of PEK-C or PES-C is through the aromatic nucleophilic substitution reaction of phenolphthalein with 4,4′-dichlorobenzophenone or 4,4′-dichlorodiphenyl sulfone in sulfolane in the presence of potassium carbonate. An alternative route to produce PEK-C and PES-C is through ring-opening polymerization of macrocyclic precursors [14,28]. In all works reported in the literature, the condensation reaction takes place in a solvent, whereas we proposed it could occur in the melted state without a solvent.

One could wonder the effect of cardo side groups on the miscibility of blends. When Scobbo [29] examined the effect of a compatibilizer on the G” peaks at the glass transition for various polymers, there was generally no change in the low *T*_g_ peak and a decrease in the maximum of the *T*_g_ peak. This trend was confirmed for our results in Figure 7, with a slight shift for the *T*_α_ of PEEK and a dramatic drop of the E” peak at *T*_α_. The conservation of the *T*_α_ for PEEK is due to the reduction of local motion imposed by the PES phase, which is stiffer, and owing to the chemical bonds created between both phases when the blend is compatibilized. The drop of the glass transition of PES stems from a faster change of a free volume with temperature in the presence of a compatibilizer.

**Figure 11:**
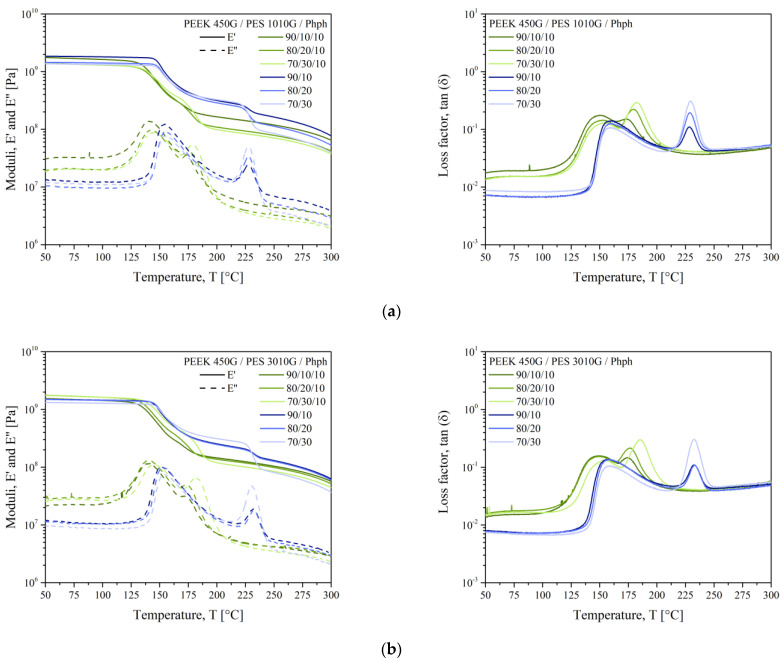
The DMTA curves of PEEK/PES blends with and without phenolphthalein: (a) 1010G; (b) 3010G.

Figure 11 superimposes the DMTA curves obtained for samples without phenolphthalein (in blue) and with phenolphthalein (in green). When comparing the curves, the T_α_ shift inward was obvious as well as the decrease of the maximum of the E” peak. Additionally, according to Scobbo [29], for a compatibilized blend, both moduli decreases after the highest glass transition. We observed the same trend, which confirmed the role of phenolphthalein as a compatibilizer. In addition, the values of E’ in the glassy state were similar for the samples with and without phenolphthalein, corroborating its role as a compatibilizer.

**Figure 12:**
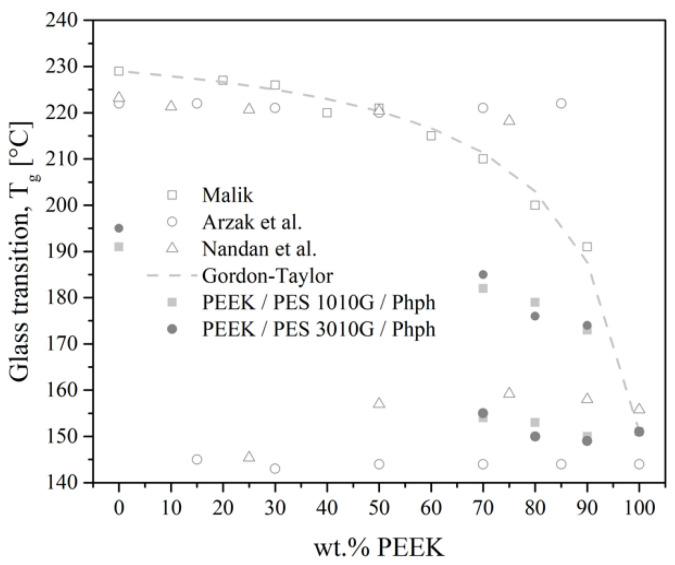
The *T*_g_ values of PEEK/PES blends versus PEEK compositions of different authors.

When considering the values of *T*_g_ obtained by other authors, they mentioned either immiscible [9,30] or partially miscible [7] blends, depending on the method of preparation. Melt-mixed blends had two *T*_g_ for a processing temperature of 360 °C [9] or 350 °C [30], while Malik [8] measured a single *T*_g_ value for melt-mixed blends at 335 °C. Their results were compared with ours processed at 375 °C, as shown in Figure 12. PEEK/PES blends may undergo a low critical soluble temperature (LCST) located between 310 and 340 °C [6]. Since the melting temperature of PEEK is around 340 °C, the PEEK-rich phase begins to flow and has higher mobility, facilitating phase separation. The high mobility of the PEEK then overcomes the specific interaction between the sulfonate functions of PES and the ether functions of PEEK.

There are several laws relating *T*_g_ to the compositions. Among them, the most frequently used for predicting *T*_g_ in a miscible polymer blend is the Gordon–Taylor equation, shown as following:

*T*_g_ = (*w*_1_*T*_g1_ + K*w*_2_*T*_g2_)/(*w*_1_ + K*w*_2_), (3)

where *T*_g,i_ is the glass transition temperature of the mixture and the components (°C), and *w*_i_ is the mass fraction (%) of the component i, and K is an adjustable fitting parameter and can be variable [31].

The latter fits well with the results of Malik [8] with the empirical parameter K equaling to 8. In other cases, the authors [9,30] have tried the Fox equation for determining the phase composition of PEEK/PES blends. However, two *T*_g_ values appeared in all compositions studied, with each of them at a temperature practically identical to that of each pure component. This indicates the presence of two virtually pure phases in the blends. In our study, the addition of phenolphthalein lowered the *T*_g_ value of the PES-rich phase. Firstly, in the 70/30 PEEK/PES blends, the glass transition of the PEEK component shifted up from 151 °C to 155 °C, and the glass transition of PES component shifted from 195 °C to 174 °C. This *T*_g_ shifting suggests that the PEEK phase is partially miscible with that of the PES component thanks to the compatibilizing effect of phenolphthalein.

#### 3.2.2. Crystallization of Blends

**Figure 13:**
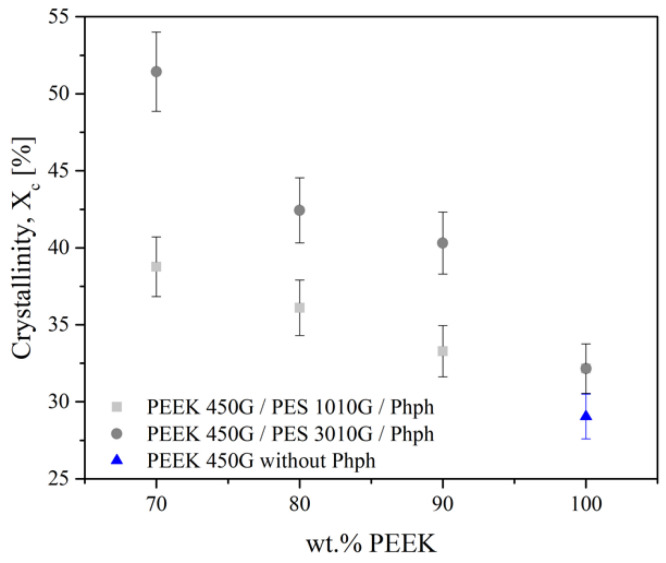
Degree of crystallinity (±5%) for blends.

In opposite to immiscible PEEK/PES blends, the degree of crystallinity of PEEK in the compatibilized blends was increased, as seen in Figure 13. The addition of phenolphthalein led to a linear increase of crystallinity with increase in the PES content. The maximum degree of crystallinity for blends with 70 wt % PES 1010G was 39%, while for 70 wt % PES 3010G it reached 51%. It means that adding phenolphthalein into blends gives more mobility to PEEK macromolecules to promote crystallization. It has to be noted that long PES chains favor the crystallization of PEEK macromolecules compared to shorter chains and the crystalline rate of PEEK blended with longer PES chains is higher than the same with shorter PES chains over 50%. It can be explained by the higher interfacial surface for the longest chains as revealed by SEM images. Indeed, the size of the droplets was smaller for the longest chains, so the surface area was higher and more PEEK chains were impacted by the mobility of PES.

We have checked that the degree of crystallinity is not impacted by the crystallization of phenolphthalein itself. For that, DSC runs have been conducted on pure phenolphthalein (see Supplementary Information). Phenolphthalein is a crystalline solid at room temperature with an orthorhombic cell. Its melting point is 260 °C, with a sharp melting peak and enthalpy equal to 150.5 J·g^−1^. The melting peak disappeared when blending PEEK and phenolphthalein, as well as PES and phenolphthalein, confirming that phenolphthalein is chemically bonded to polymeric chains in the melted state. During the cooling and second heating scan of pure phenolphthalein, the melting peak disappeared and a glass transition temperature was visible at around 80 °C. One assumes structural changes of phenolphthalein with temperature. Its thermal stability has been measured by TGA experiments (see Supplementary Information). No evidence of degradation was noticed before reaching 330 °C, where the phenolphthalein lost 5% of its mass at a heating ramp of 10 °C·min^−1^ in air.

Therefore, the PES-rich phase is softer due to the phenolphthalein, so that the PEEK-rich phase is less constrained than in pure PEEK. The increase in the crystallization rate of PEEK is attributed to this mobility gain.

#### 3.2.3. Morphological Analysis by SEM

**Figure 14:**
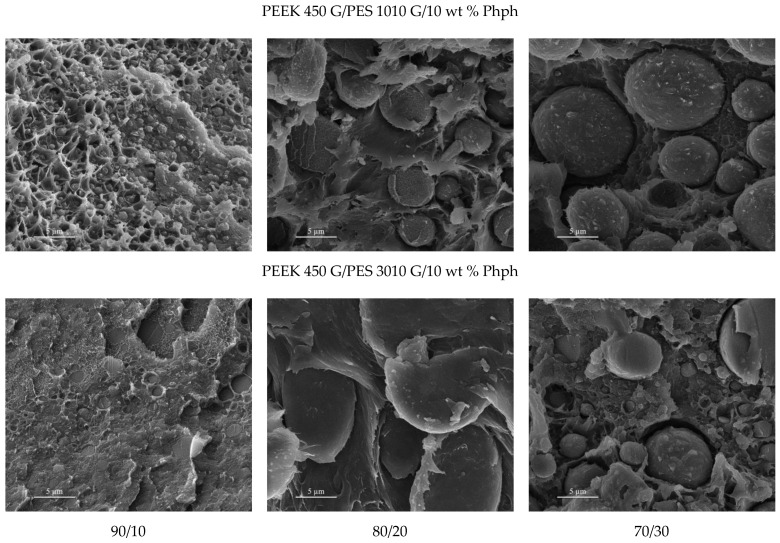
The SEM images of the microstructures of PEEK and PES blends (wt %) with phenolphthalein.

The morphologies of compatibilized blends are shown in Figure 14. The SEM images revealed droplets attributed to the shift from a PES-rich phase into a continuous PEEK-rich phase. When focusing on PES surfaces, the spherical domains are surrounded by some particles or a small piece of polymer that look stacked on it, which indicates an improvement of the interfacial adhesion.

The PES droplet size increased with PES concentration. The size of PES droplets was around 1 µm for blends with 10 wt % PES, while with the increase of PES content, the size was around 5 µm for 20 wt % PES and even over 10 µm for 30 wt % PES. The same concentration of phenolphthalein was added in all blends. Let us assume that in all blends, we have the same phenolphthalein concentration at the interface. Since the amount of phenolphthalein is constant, the surface can be covered is the same. When the PES volume amount is doubled, the droplet size is doubled to keep the same covered surface. In this case, one could expect to double the diameter of the droplet when the amount of PES is doubled. Indeed, this is what we seem to observe in the SEM pictures: in the 90/10/10 blend, the droplets sizes were about 1 µm in diameter. When the PES amount was doubled, such as the 80/20/10 blend, the droplets sizes observed were about 5 µm in diameter. In the case of 30 wt % PES, the droplets sizes were about 5–15 µm in diameter at most. The quantitative trends are similar for both PES grades.

In addition, the size of the droplets was smaller for PES 3010G compared to that for PES 1010G. This could be due to the difference in viscosity: it requires a higher shear rate to get uniformly dispersed phases when the viscosity of each component is far from one to the other.

**Figure 15:**
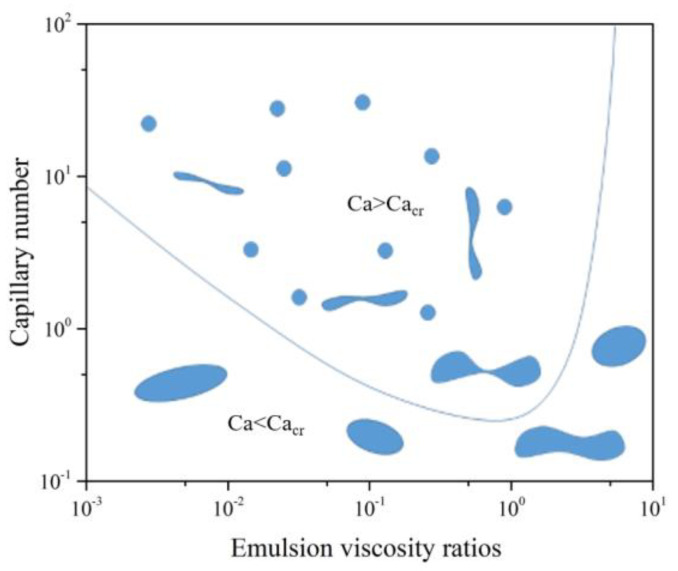
Relation between critical capillary number and viscosity ratio in polymer blends.

Indeed, the viscosity ratio affects the size of the droplets according to the relationship proposed by Grace in 1982 [32]. The dependency of the capillary number with the viscosity ratio λ is displayed in Figure 15.

The capillary number was obtained from the following equation:

Ca = ηγD/Г(4)

where η is the viscosity of the continuous phase (m·Pa·s), γ is the shear rate (rad·s^−1^), D is the droplet size (m), and Г is the interfacial tension (N·m^−1^). For a value of viscosity ratio, droplets are broken into smaller droplets, when the viscous forces are higher than capillary forces, meaning Ca > Ca_cr_. As illustrated in Figure 15, the curve separates two domains with the area above the line representing the droplets are broken into smaller ones. We will also note that the curve passes through a minimum when the viscosity ratio is about unity. Below the line, the droplets cannot be divided into smaller ones. The critical capillary number will increase when the gap between viscosities is large.

The viscosity ratios were calculated at 0.063 for PES 1010G/PEEK and 0.3 for PES 3010G/PEEK. Hence, the Ca_cr_ was lower for PES 3010G/PEEK blend. Assuming the shear rate, the viscosity of the main phase (PEEK) and the interfacial tension were identical, and the emulsification was deeper in the break-up zone of Grace’s diagram. One can reasonably expect to have smaller diameter droplets for this blend. 

Increasing the shear rate during the blending process of this ternary system is a promising option to create smaller droplets. Indeed, the best resistance to fracture is usually obtained with the smallest diameter droplets.

## 4. Conclusions

The main goal was to elaborate a new high-performance thermoplastic material while keeping high mechanical strength at the glassy state. In polymer blends, the miscibility of the two polymers is often required, especially in load-carrying applications, since low miscibility gives a weak interfacial adhesion, leading to a poor stress transfer from one to the other phase and up to fracture.

PEEK has been blended with polyethersulfone at the melted state. The resulting PEEK/PES blends were immiscible. Two glass transition temperatures were measured by DSC and DMTA, and the crystallinity rate was unchanged, showing that the PES phase had no impact on the PEEK crystalline phase. Two grades of PES have been compared, and there was no effect of chain length.

When adding phenolphthalein to PEEK/PES blends, the glass transitions were shifted inward, as an indication of miscibility. The rheological curves and the analysis of the morphology of blends demonstrated that phenolphthalein acted as a compatibilizer. We suggested that phenolphthalein creates cardo side groups on PEEK and PES chains by nucleophilic substitution in the melted state, although these condensation reactions were reported only in a solvent at a lower temperature until now. These cardo side groups increased the miscibility of PEEK and PES. Due to aromatic moieties in phenolphthalein, the PEEK and PES chains were extended, but their storage moduli in the glassy region were kept identical as pure PEEK.

In addition, the effect of phenolphthalein was stronger on *T*_g_ of PES, showing that phenolphthalein acts as a plasticizer for PES by increasing the free volume to favor local motion.

Despite cardo side groups chemically bonded to PEEK chains, the mobility of the PEEK phase favored its crystallization. Moreover, the crystalline rate of PEEK was increased with PES content in ternary blends. Thus, the PES-rich phase softened the PEEK phase to give macromolecules more mobility to self-organization into crystalline structures. Still, the effects of PES chain length on miscibility and morphology are not conclusive.

The morphological analysis displayed nano- to microsized PES spherical domains. Further work will confirm that the mechanical properties are improved by phenolphthalein through a size reduction of the dispersed PES domains and an increase of the interfacial adhesion. This work is a step to inspire materials with improved miscibility and mechanical properties that could be processed by injection molding, extrusion, or additive manufacturing.

## Figures and Tables

**Table 1:** Properties of the blend components.

Polymer	Melt Flow Index (g·(10 min)^−1^)	Molecular Weight (g·mol^−1^)	Shear Viscosity (Pa·s)	Density (g·cm^−3^)
PEEK 450G	5	98,000 [16]	5000	1.30
Ultrason E1010	150	35,000 [17]	300	1.37
Ultrason E3010	35	58,000 [17]	1500	1.37

**Table 2:** Compositions of the samples used.

	Polyetheretherketone (PEEK)	Polyethersulfone (PES)		Phenolphthalein
	450G	1010G	3010G	Phph
	(wt %)	(wt %)	(wt %)	(wt %)
vPEEK	100			
vPES		100	100	
PEEK/PES	100	0	-	
	90	10	-	
	80	20	-	
	70	30	-	
	0	100	-	
	100	-	0	
	90	-	10	
	80	-	20	
	70	-	30	
	0	-	100	
PEEK/PES/Phph	100	0	-	10
	90	10	-	10
	80	20	-	10
	70	30	-	10
	0	100	-	10
	100	-	0	10
	90	-	10	10
	80	-	20	10
	70	-	30	10
	0	-	100	10

**Table 3:** Glass transition temperatures of blends.

	PEEK/PES Blends	Glass Transition of PEEK (°C)	Glass Transition of PES (°C)
	vPEEK	147	
PEEK 450G/ PES 1010G	100/0	159	-
	90/10	159	228
	80/20	161	229
	70/30	160	230
	0/100	-	228
	vPES		232
	vPEEK	147	
PEEK 450G/ PES 3010G	100/0	159	-
	90/10	158	233
	80/20	159	233
	70/30	158	233
	0/100	-	223
	vPES		236

**Table 4:** Glass transition temperatures of blends with phenolphthalein.

	PEEK/PES/Phph Blends	Glass Transition of PEEK (°C)	Glass Transition of PES (°C)
PEEK 450G/ PES 1010G/ Phph	100/0/10	151	-
	90/10/10	150	173
	80/20/10	153	179
	70/30/10	154	182
	0/100/10	-	191
PEEK 450G/ PES 3010G/ Phph	100/0/10	151	-
	90/10/10	149	174
	80/20/10	150	176
	70/30/10	155	185
	0/100/10	-	195

## Data Availability

The data presented in this study are available on request from the corresponding author.

## Supplementary Materials

The following are available online at https://www.mdpi.com/article/10.3390/polym13091466/s1, Figure S1: Enlarged DSC thermograms on glass transition temperature for PEEK/PES 1010G blends (a) and PEEK/PES 3010G blends (b), Figure S2: Enlarged DSC thermograms on glass transition temperature for PEEK/PES 1010G blends with phenolphthalein (a) and PEEK/PES 3010G blends with phenolphthalein (b), Figure S3: DSC thermograms of pure PEEK with and without Phph (a) and pure PES with and without Phph (b,c), Figure S4: Glass transition of PES (a) and PEEK (b,c) versus Phenolphthalein composition, Figure S5: FTIR spectra of pure polymer with and without Phph, Figure S6: DSC thermogram of phenolphthalein, Figure S7: Thermal resistance of phenolphthalein by TGA.
