# Supplementary material for: Poly(etheretherketone)/Poly(ethersulfone) Blends with Phenolphthalein: Miscibility, Thermomechanical Properties, Crystallization and Morphology
Source: Polymers (Basel). 2021 May 1;13(9):1466. doi: 10.3390/polym13091466 (PMC8124672; doi:10.3390/polym13091466)
Supplement: Supplementary file 1 [file polymers-13-01466-s001.zip › polymers-1197301-supplementary.pdf]

Article

Poly(etheretherketone) / Poly(ethersulfone) Blends with Phenolphthalein: Miscibility, Thermomechanical Properties, Crystallization and Morphology

Adrian Korycki [1,2], Christian Garnier [1], Amandine Abadie [1], Valerie Nassiet [1], Tarek Sultan [2] and France Chabert [1,*]

[1] LGP-ENIT-INPT, Université de Toulouse, 47 avenue d'Azereix, 65016 Tarbes cedex (France)
[2] Prismadd Montauban, 2 bis rue Georges Courteline, 82000 Montauban (France)
* Correspondence: france.chabert@enit.fr

List of supporting information:

Figure S1. Zoom of DSC thermogram on glass transition temperature for: (**a**) PEEK/PES 1010G blends; (**b**) PEEK/PES 3010G blends

Figure S2. Zoom of DSC thermogram on glass transition temperature for: (**a**) PEEK/PES 1010G blends; (**b**) PEEK/PES 3010G blends, with phenolphthalein

Figure S3. DSC thermograms of pure: (**a**) PEEK; (**b**,**c**) PES, with and without Phph

Figure S4. Glass transition of: (**a**) PES; (**b**,**c**) PEEK, versus Phenolphthalein composition

Figure S5. FTIR spectra of pure polymer with and without Phph

Figure S6. DSC thermogram of phenolphthalein

Figure S7. Thermal resistance of phenolphthalein by TGA

Supplementary 1 (Figure 2):

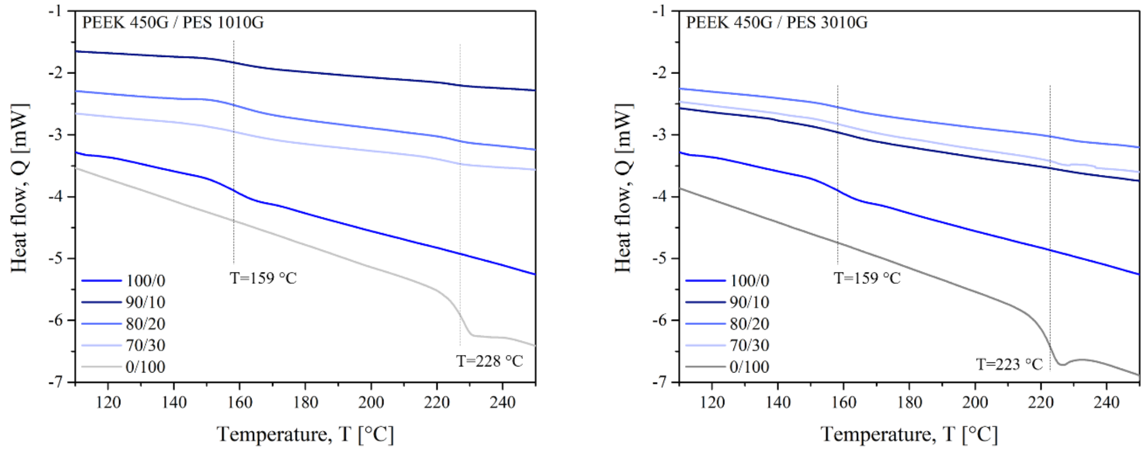

(a) (b)

Figure S1. Zoom of DSC thermogram on glass transition temperature for: (**a**) PEEK/PES 1010G blends; (**b**) PEEK/PES 3010G blends

Supplementary 2 (Figure 6):

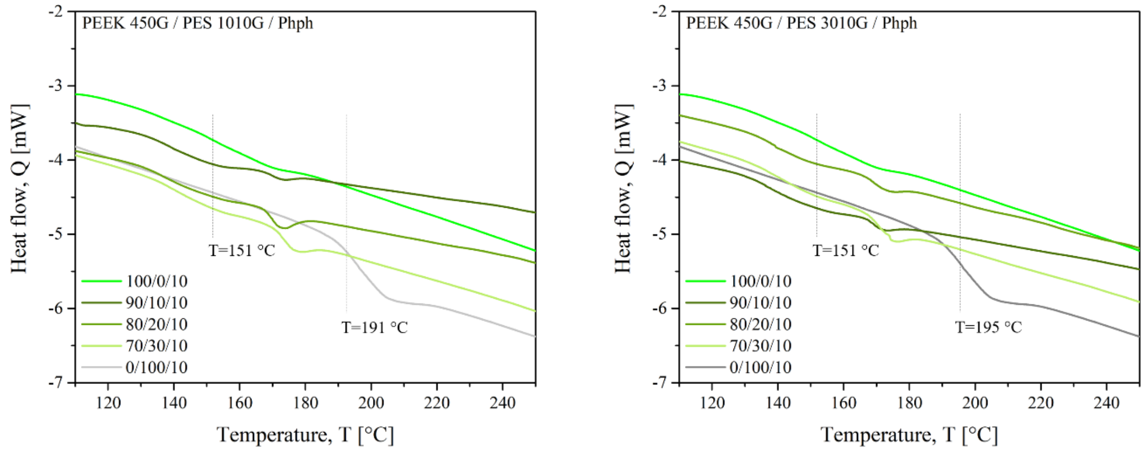

(a) (b)

Figure S2. Zoom of DSC thermogram on glass transition temperature for: (**a**) PEEK/PES 1010G blends; (**b**) PEEK/PES 3010G blends, with phenolphthalein

Supplementary 3 (Figure 6):

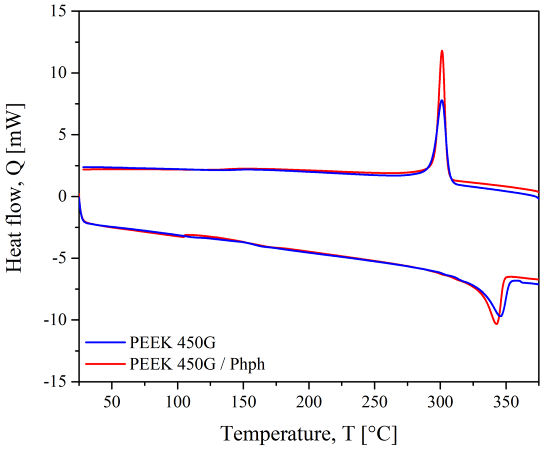

(a)

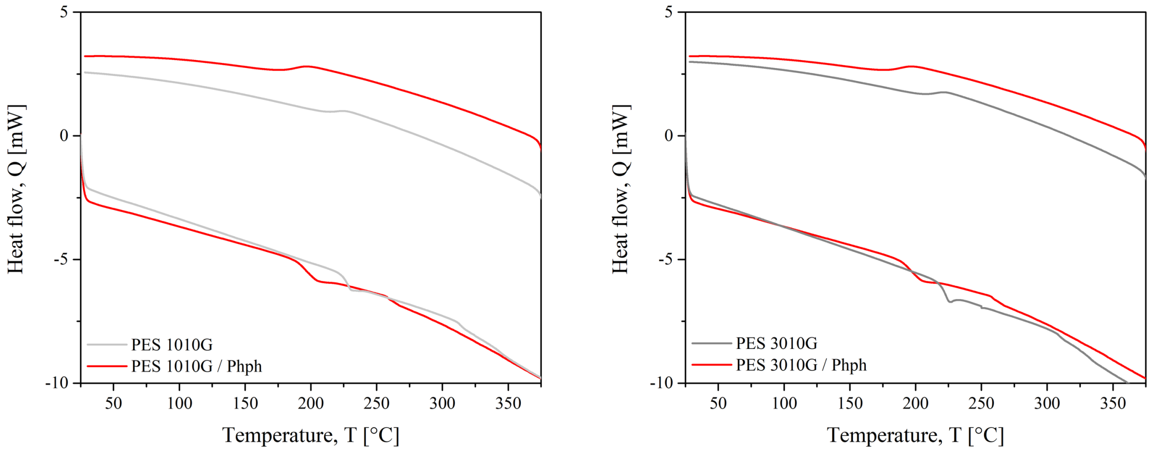

(**b**) (**c**)

Figure S3. DSC thermograms of pure: (**a**) PEEK; (**b**,**c**) PES, with and without Phph

Supplementary 4 (Figure 7):

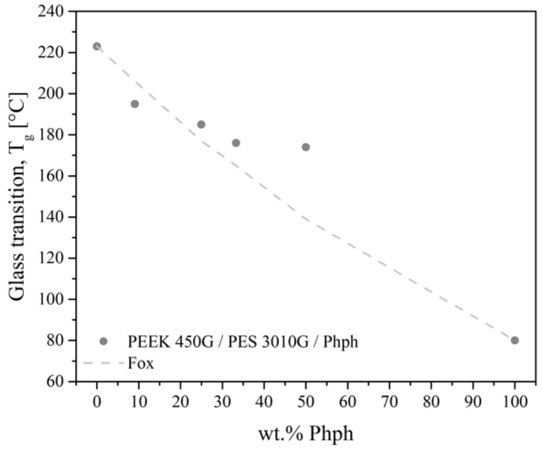

(**a**)

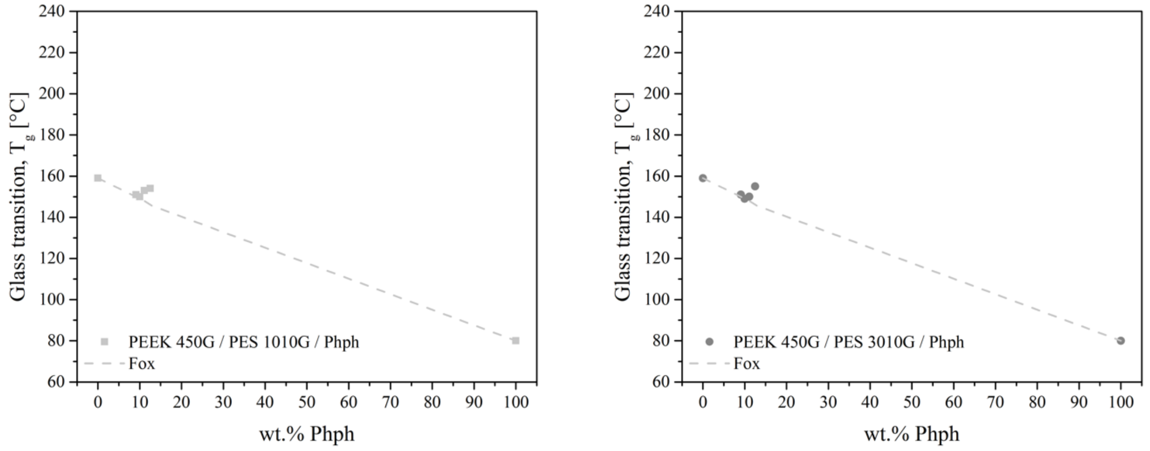

(**b**) (**c**)

Figure S4. Glass transition of: (**a**) PES; (**b**,**c**) PEEK, versus Phenolphthalein composition

Supplementary 5 (Figure 9):

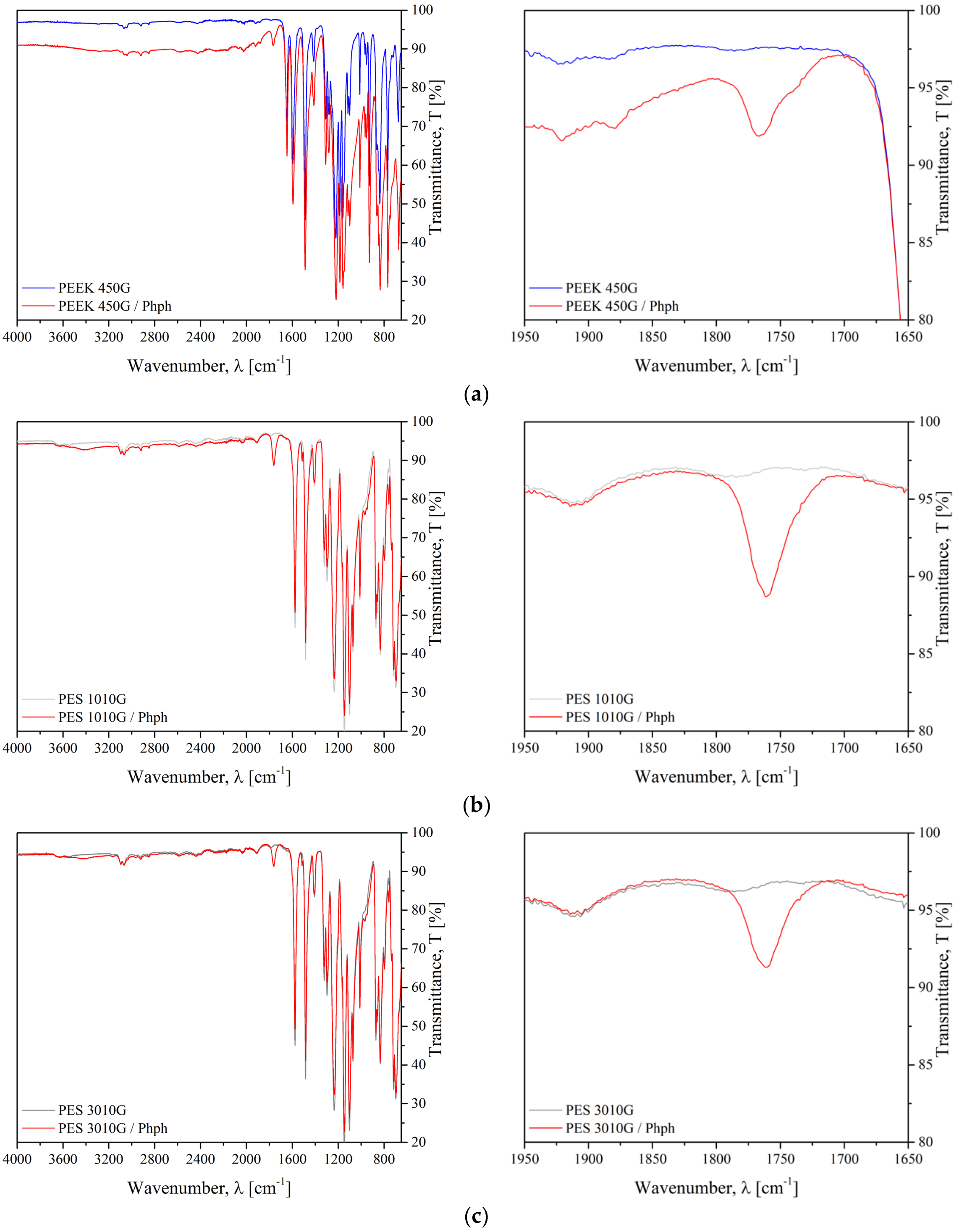

Figure S5. FTIR spectra of pure polymer with and without Phph

Supplementary 6 (Figure 13):

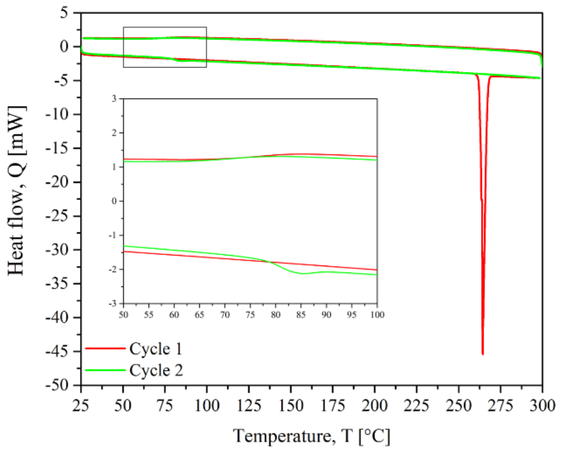

Figure S6. DSC thermogram of phenolphthalein

Supplementary 7 (Figure 13):

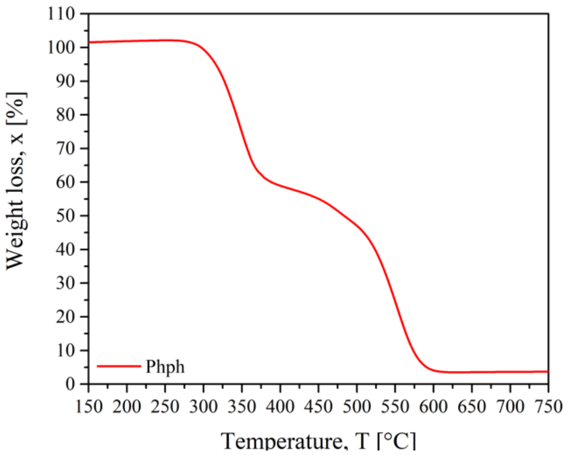

Figure S7. Thermal resistance of phenolphthalein by TGA
